# Unraveling FATP1, regulated by ER-β, as a targeted breast cancer innovative therapy

**DOI:** 10.1038/s41598-019-50531-3

**Published:** 2019-10-01

**Authors:** Cindy Mendes, Filipa Lopes-Coelho, Cristiano Ramos, Filipa Martins, Inês Santos, Armanda Rodrigues, Fernanda Silva, Saudade André, Jacinta Serpa

**Affiliations:** 1 CEDOC, Chronic Diseases Research Centre, NOVA Medical School|Faculdade de Ciências Médicas, Universidade NOVA de Lisboa, Campo dos Mártires da Pátria, 130, 1169-056 Lisboa, Portugal; 2 Instituto Português de Oncologia de Lisboa Francisco Gentil (IPOLFG), Rua Prof Lima Basto, 1099-023 Lisboa, Portugal

**Keywords:** Prognostic markers, Breast cancer

## Abstract

The biochemical demands associated with tumor proliferation prompt neoplastic cells to augment the import of nutrients to sustain their survival and fuel cell growth, with a consequent metabolic remodeling. Fatty acids (FA) are crucial in this process, since they have a dual role as energetic coins and building blocks. Recently, our team has shown that FATP1 has a pivotal role in FA transfer between breast cancer cells (BCCs) and non-cancerous cells in the microenvironment. We aimed to investigate the role of FATP1 in BCCs and also to explore if FATP1 inhibition is a promising therapeutic strategy. In patients’ data, we showed a higher expression of *FATP1/SLC27A1* in TNBC, which correlated with a significant decreased overall survival (OS). *In vitro*, we verified that FA and estradiol stimulated *FATP1/SLC27A1* expression in BCCs. Additionally, experiments with estradiol and PHTPP (ER-β antagonist) showed that estrogen receptor-β (ER-β) regulates *FATP1/SLC27A1* expression, the uptake of FA and cell viability, in four BCC lines. Furthermore, the inhibition of FATP1 with arylpiperazine 5k (DS22420314) interfered with the uptake of FA and cell viability. Our study, unraveled FATP1 as a putative therapeutic target in breast cancer (BC).

## Introduction

Breast cancer (BC) is the most frequent malignant neoplasia in women worldwide, with an estimated 1.7 million cases and 521,900 deaths in 2012, accounting for 25% of all cancer cases and 15% of all cancer deaths among females^1^. The tumor microenvironment is well recognized to play a major role in the development and progression of cancer^2^. The non-neoplastic cells of the tumor microenvironment including fibroblasts, adipocytes, immune and endothelial cells seem to be determinant in cancer biology since they act as a functional network in which soluble factors and organic molecules are transiently shared^3^. Cancer associated fibroblasts (CAFS) may be the predominant cellular component of the tumor microenvironment and are known to express aromatase, a key enzyme in estrogen synthesis, resulting in the production of intra-tumoral estrogen frequently observed in BC^4^. Moreover, besides being essential for normal growth and differentiation in the mammary gland, estrogen plays an important role in the development and progression of BC^5^. Estrogen action is mediated by binding to estrogen receptor (ER)-α and/or ER-β which act as transcription factors^6^. Although encoded by different genes, both isoforms show similar functional and structural characteristics, interacting similarly with endogenous estrogens, mainly 17β-estradiol^7^. The classification of ER positive and ER negative BCs is based only on the presence of ER-α in their nuclei^8^, without considering the expression of ER-β. Accordingly, therapies that interfere with estrogen signaling such as estrogen antagonists (tamoxifen) and aromatase inhibitors have been developed and clinically implemented for the treatment of ER-positive BC^9^. However, these drugs have unwanted side effects in non-target tissues and some carcinomas become resistant^10^. Presently, only ER-α has been used in a clinical setting, since its protein levels are elevated in BCs compared with normal tissue, while ER-β is being more disregarded. ER-β mRNA was reported to be significantly upregulated in tamoxifen-resistant BCs compared with tamoxifen-sensitive tumors, suggesting a link between ER-β overexpression and tamoxifen resistance^11^. ER-β prognostic and predictive value remains controversial, however, targeting this receptor in some cases could offer new treatment options for BC patients^12^. Compared with hormone receptor-positive BCs, triple-negative BCs (TNBC) are associated with a worse prognosis and more aggressive phenotypes^13^. Since patients with TNBC do not benefit from hormonal based therapies, there is a great need to identify treatment options.

To fulfill the biosynthetic demands associated with proliferation and consequent tumor growth, cells must increase the import of nutrients that is supported and supports a metabolic remodeling^14^. In a proliferative tumor niche, fatty acids (FA) are crucial since they are both fuel and construction blocks, maintaining cell renewal and division^15,16^.

Conceptually, adipocytes are the main suppliers of FA, however, we previously reported that cancer associated fibroblasts (CAFs) also function as hubs of FA to supply the needs of cancer cells^15^. Breast cancer cells (BCCs) exposed to CAFs-conditioned media increased their lipid uptake and the expression of *FATP1/SLC27A1* (FA transport protein 1), promoting FA transfer. FATP1 is an integral membrane protein known to enhance the uptake of long-chain and very long-chain FA into cells^17^. Considering FA transfer from CAFs to BCCs, FATP1 appears to be a suitable candidate to treat BC and a possible marker of disease outcome.

In the present study, we further investigated the role of FATP1 in breast cancer cells (BCCs) survival and behavior and explored a new therapeutic strategy. We demonstrated that FA and estradiol stimulate *FATP1/SLC27A1* expression and we unraveled the crucial role of ER-β in *FATP1/SLC27A1* regulation and modulation in the uptake of FA. Our *in vitro* findings were supported by patients’ data showing a higher expression of *FATP1/SLC27A1* in more aggressive and invasive BCs. Furthermore, the inhibition of FATP1 with arylpiperazine 5k (DS22420314) interfered with the uptake of FA and cell proliferation, validating the importance of FATP1 as a putative therapeutic target in BC.

## Results

### *FATP1/SLC27A1* expression is associated to triple negative BC (TNBC) and correlates with lower overall survival (OS) and relapse free survival (RFS) times

**Figure 1:**
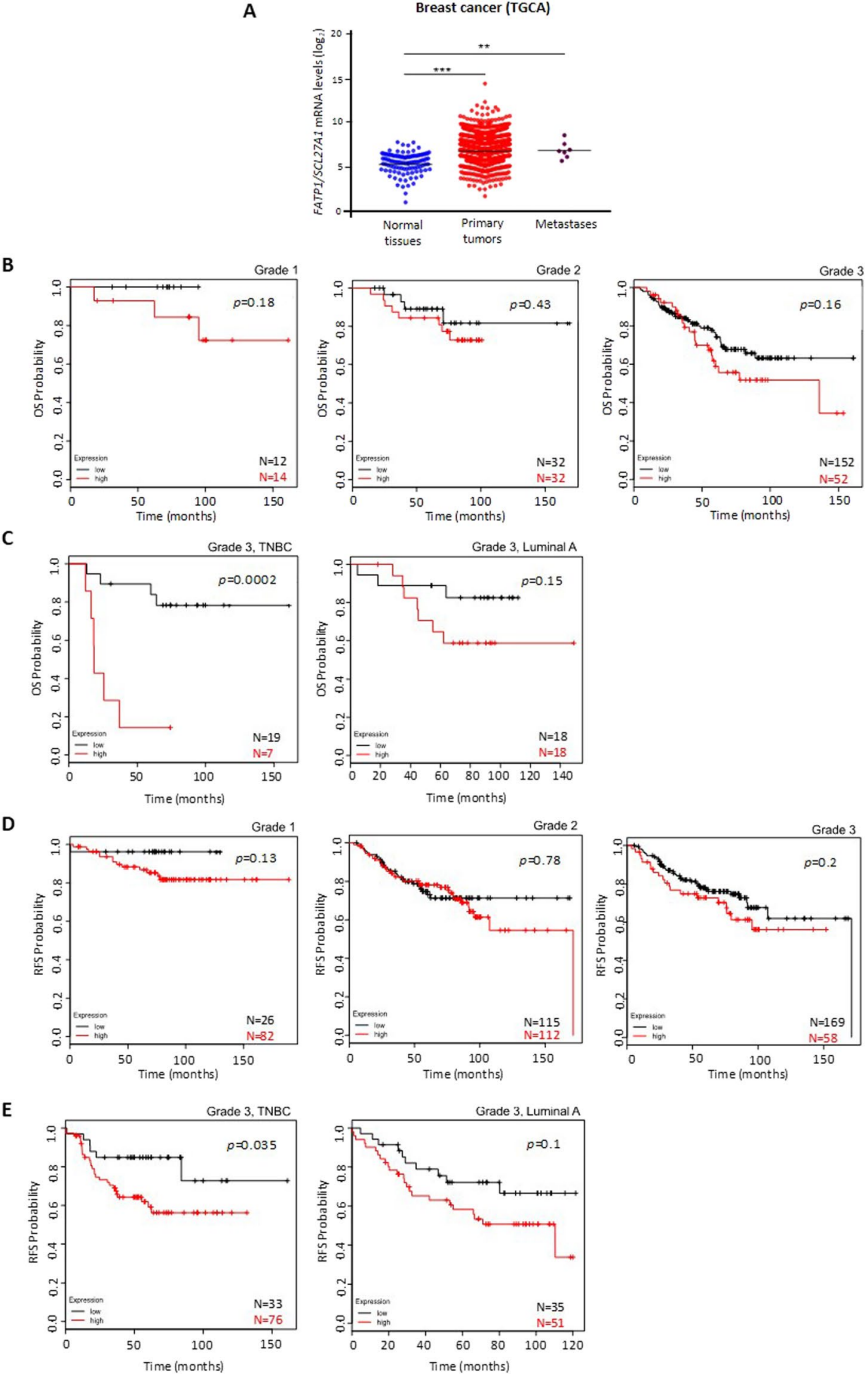
Patients with higher levels of *FATP1/SLC27A1* expression have a lower OS and RFS. (**A**) Analysis of *FATP1/SLC27A1* expression profiles in normal breast tissue, primary carcinomas and metastasis, data extracted from the TCGA database. A two-tailed unpaired Student’s t-test with Welch’s correction was used. Comparison of the overall survival (OS) and relapse free survival (RFS) curves of GEO database patients with high levels of FATP1 (red line) and low levels of FATP1 (black line) expression using Kaplan-Meier method. (**B**) Kaplan-Meier survival curves for patients with BC grade 1 (n = 26), grade 2 (n = 64) and grade 3 (n = 204). (**C**) Kaplan-Meier survival curves for patients with grade 3, TNBC BC (n = 26) and grade 3, luminal A BC (n = 36). (**D**) Kaplan-Meier RFS curves for patients with BC grade 1 (n = 108), grade 2 (n = 227) and grade 3 (n = 227). (**E**) Kaplan-Meier survival curves for patients with grade 3, TNBC BC (n = 108) and grade 3, luminal A BC (n = 86).

At first, to understand the impact of *FATP1/SLC27A1*expression in BC patients, we analyzed data extracted from the TCGA database. Considering BC patients and the expression of *FATP1/SLC27A1*, it was shown that BCs and metastasis express higher levels of *FATP1/SLC27A1* than normal breast tissue (Fig. 1A).

To determine the relevance of the expression levels of the *FATP1/SLC27A1* gene on the clinical outcome of BC patients, a Kaplan-Meier Plotter analysis^18^ was employed, using data from Gene Expression Omnibus (GEO) database. Overall survival (OS) in BC patients was determined in grade 1 (N = 26), grade 2 (N = 64) and grade 3 (N = 204), with high (red line) and low (black line) expression of *FATP1/SLC27A1*. Despite being not significant, a trend in decreased OS was observed with high *FATP1/SLC27A1* expression and increased tumor grade (Fig. 1B). Looking at two different subtypes of grade 3 BCs: TNBC (N = 26) and luminal A (N = 36), with respectively high and low levels of *FATP1/SLC27A1*; the OS was significantly lower in patients with carcinomas with high levels of *FATP1/SLC27A1* (Fig. 1C).

Relapse free survival (RFS) time analysis was assessed in grade 1 (N = 108), grade 2 (N = 227) and grade 3 (N = 227) BC patients with high and low expression of *FATP1/SLC27A1* (Fig. 1D) and also at the two different subgroups of grade 3 tumors: TNBC and luminal A. Patients with grade 3 disease and with high expression of *FATP1/SLC27A1* showed a lower RFS comparing with grade 1 and 2 patients. As shown in Fig. 1E, grade 3 BC patients, with a TNBC (109 patients), with high *FATP1/SLC27A1* expression (red line) displayed a significantly lower RFS than patients with low *FATP1/SLC27A1* expression (black line). A similar observation was registered for grade 3, luminal A patients (86 patients) but no statistical significance was obtained.

In breast tumor sections, the expression profile of FATP1 protein showed a statistically significant (*p* = 0.015) association with TNBC subtype of BC (Table 1).

**Table 1:** Immunohistochemical analysis of FATP1 expression in BC sections.

	FATP1 positivity	*p* value
TNBC	65% (13/20)	0.015
Luminal A	26.3% (5/19)	

The expression of FATP1 positivity is associated with triple negative BC (TNBC; *p* = 0.015) but not with luminal A type of BC. Immunohistochemical data was analyzed using univariate analysis (two-tailed t-test) on SPSS software.

### Linoleic acid (C18) and estradiol stimulate FATP1 expression and estradiol stimulates the binding of ER-β to *FATP1/SLC27A1* promoter

Afterwards, we intended to evaluate the modulation of *FATP1/SLC27A1* expression in BCCs. The first screening was performed in MDA-MB-231, which is a TNBC cell line, representing the tumors in which the outcome in the clinical setting was worst and correlated with high expression of *FATP1/SLC27A1*. As we previously described, FATP1 mediates the uptake of FA by cancer cells^15^ and because the promoter region of *FATP1/SLC27A1* gene has estrogen responsive elements (ERE), we tested the effect of linoleic acid (C18) and estradiol in *FATP1/SLC27A1* expression. The TNBC molecular subtype is characterized also by the lack of estrogen receptor, however this characterization is performed only based on ER-α expression, ignoring the ER-β. That is why we thought it would be worth testing the effect of estradiol in *FATP1/SLC27A1* expression.

**Figure 2:**
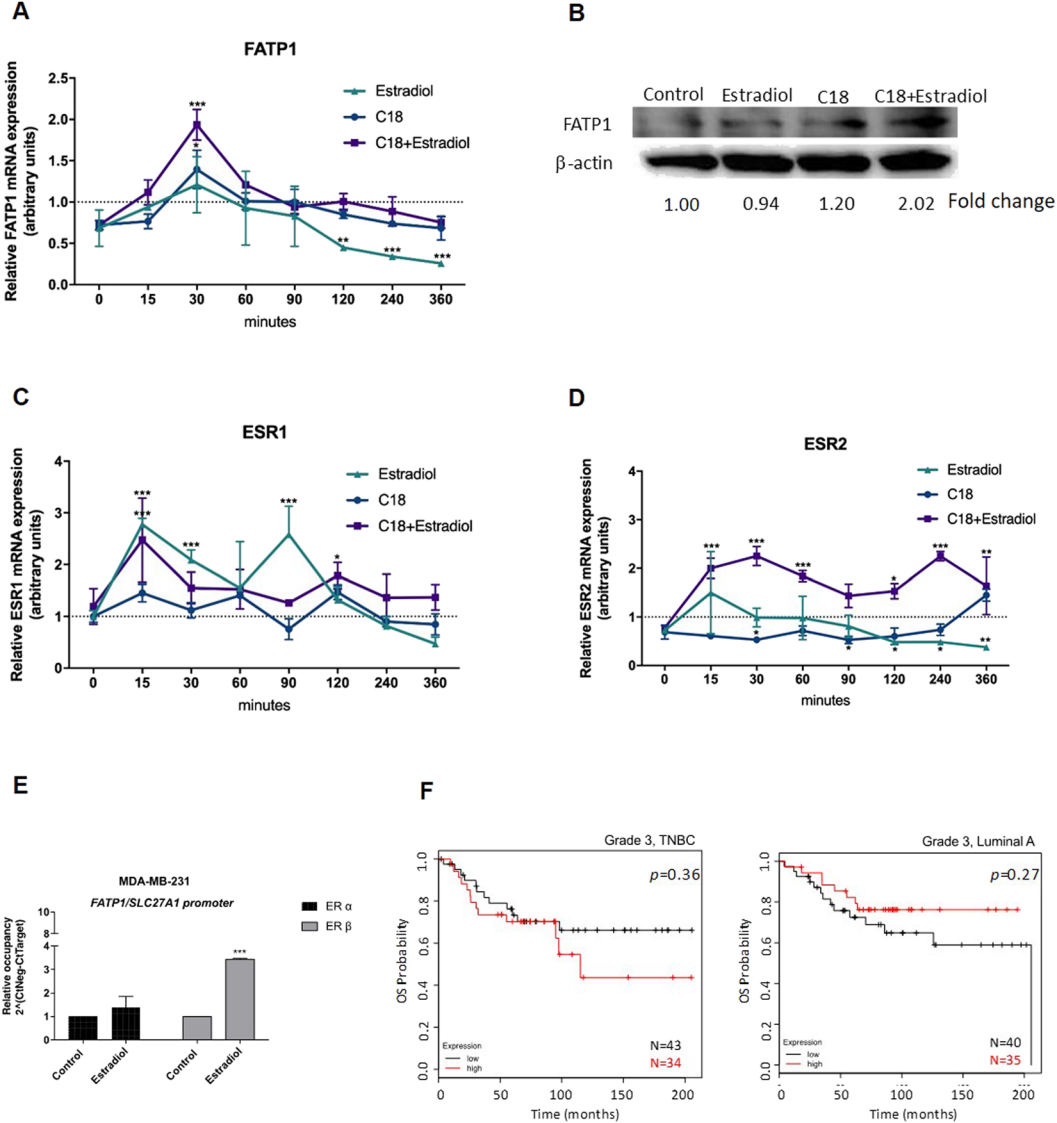
Linoleic acid (C18) and estradiol stimulate *FATP1/SLC27A1* expression and estradiol stimulates the binding of ER-β to *FATP1/SLC27A1* promoter (**A**) *FATP1/SLC27A1*, (**C**) *ESR1* and (**D**) *ESR2* expression levels in MDA-MB-231 cells in a pulse chase experiment referenced to the control condition. (**B**) Western blotting for FATP1 detection, the numbers are indicative of fold change of each condition (normalized for the respective β-actin) in relation to control. (**C**) *ESR1* and (**D**) *ESR2* expression levels in MDA-MB-231 cells in a pulse chase experiment referenced to the control condition. In relative qPCR, hypoxanthine-guanine phosphoribosyltransferase (HPRT) gene was used as housekeeping gene. (**E**) Relative occupancy of ER-α and ER-β at *FATP1/SLC27A1* promoter. Cells were cultured in control and in estradiol conditions and ER-α and ER-β binding was assessed by ChIP. Data are mean ± error bars of biological triplicates, only adherent cells were analyzed, dead cells in culture media were discarded. (**F**) Kaplan-Meier survival curves for patients with grade 3, TNBC (n = 77) and grade 3, luminal A BC (n = 75) for the *ESR2* gene. *p < 0.05 **p < 0.01 ***p < 0.001. (*) represents the statistical analysis in relation to control condition (dot line). For pulse chase qPCR experiments a Two-way ANOVA with Tukey’s test was used.

In order to evaluate the modulation of *FATP1/SLC27A1*, MDA-MB-231 cells were exposed to C18 and estradiol, in separate and in combination. FATP1 mRNA levels were significantly higher in MDA-MB-231 cells after 30 min of stimulation with C18 and C18 plus estradiol (Fig. 2A), indicating that cell lines benefit from *FATP1/SLC27A1* expression to cope with the increased levels of C18. The significant decrease of *FATP1/SLC27A1* mRNA in cells exposed only to estradiol shows that, without C18 boost, the stimulation of *FATP1/SLC27A1* expression by estradiol is not required in this cell line. At the protein levels and reinforcing the role of estradiol and C18 on *FATP1/SLC27A1* expression, FATP1 protein levels increase upon C18 and C18 plus estradiol stimuli in a much longer timepoint of 16 h (Fig. 2B).

As aforementioned, *FATP1/SLC27A1* gene has ERE sequences in its promoter region, so we also evaluated the pulse chase dynamics of *ESR1* (encodes ER-α) and *ESR2* (encodes ER-β) genes expression. It was very interesting to see that the expression of *ESR2* was significantly higher in cells exposed to C18 + estradiol, in all timepoints (Fig. 2D), whereas *ESR1* showed a significant up and down expression in a few timepoints and related to estradiol exposure (Fig. 2C).

In order to find a functional link between ER-α or ER-β with the regulation of *FATP1/SLC27A1* gene expression, a ChIP assay was performed. The relative occupancy of ER-β in *FATP1/SLC27A1* promoter significantly increased upon estradiol stimulation (Fig. 2E). We observed no differences in the relative occupancy of ER-α in the *FATP1/SLC27A1* promoter.

Regarding *ESR2* (encoding ER-β) expression in the clinical setting, patients with TNBC (N = 77) with high levels of *ESR2* expression showed a lower survival rate, although this association was not significant. The opposite was observed in luminal A group (Fig. 2F).

### *FATP1/SLC27A1* expression is regulated by ER-β

To confirm the role of ER-β on *FATP1/SLC27A1* expression, we assessed FATP1 levels by flow cytometry upon the exposure to an ER-β antagonist (PHTPP) with estradiol and/or C18. For that, we used MDA-MB-231 and also MCF7, a luminal A cell line. In order to exclude the interference of ER-α, cells were also tested with MPP and Fulvestrant, antagonists of ER-α. The concentrations of ER-α and ER-β antagonists and agonists were selected from the literature^19–21^.

**Figure 3:**
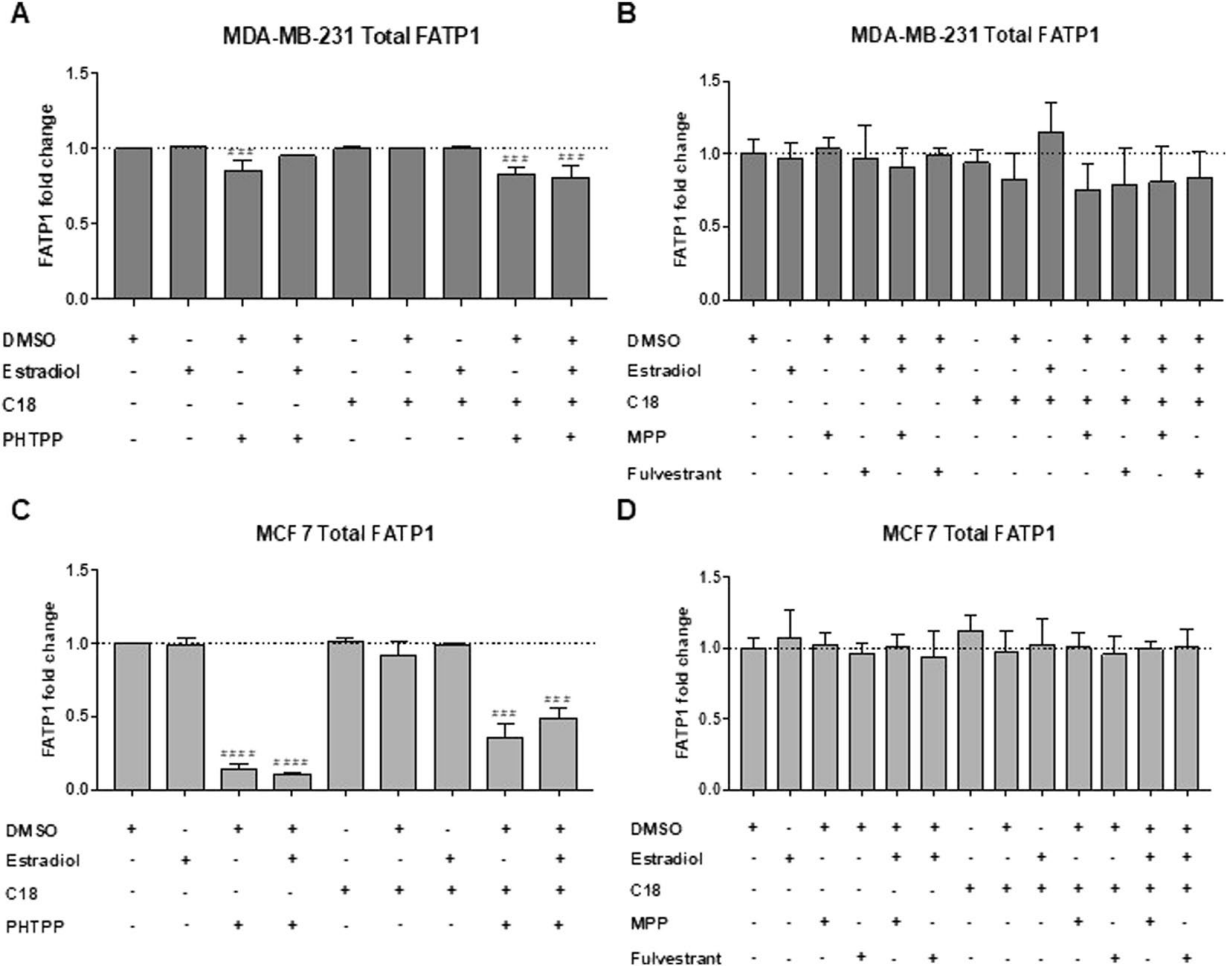
FATP1 is downregulated in BCCs treated with PHTPP. BCCs were cultured in control (baseline culture medium represented by the dot line), control DMSO, estradiol and/or C18 conditions, in the presence or absence of PHTPP (**A**,**C**) and in the presence or absence of MPP and Fulvestrant (**B**,**D**), being analyzed by flow cytometry. Values are referenced to control/DMSO conditions within each cell line. Biological triplicates were tested, only adherent cells were analyzed and dead cells in culture media were discarded. Results are shown as mean ± SD. *p < 0.05, **p < 0.01, ***p < 0.001, ****p < 0.0001. (*) represents the statistical analysis in relation to control condition and (#) represents the statistical analysis in relation to DMSO condition. One-way ANOVA with Dunnett’s multiple comparisons test.

The amount of total FATP1 positive cells was significantly lower when MDA-MB-231 and MCF7 cells were treated with the ER-β antagonist (PHTPP) (Fig. 3A,C). MPP and Fulvestrant (ER-α antagonists) showed non-significant changes in FATP1 levels (Fig. 3B,D).

### Breast cancer (BC) FA uptake is regulated by ER-β

**Figure 4:**
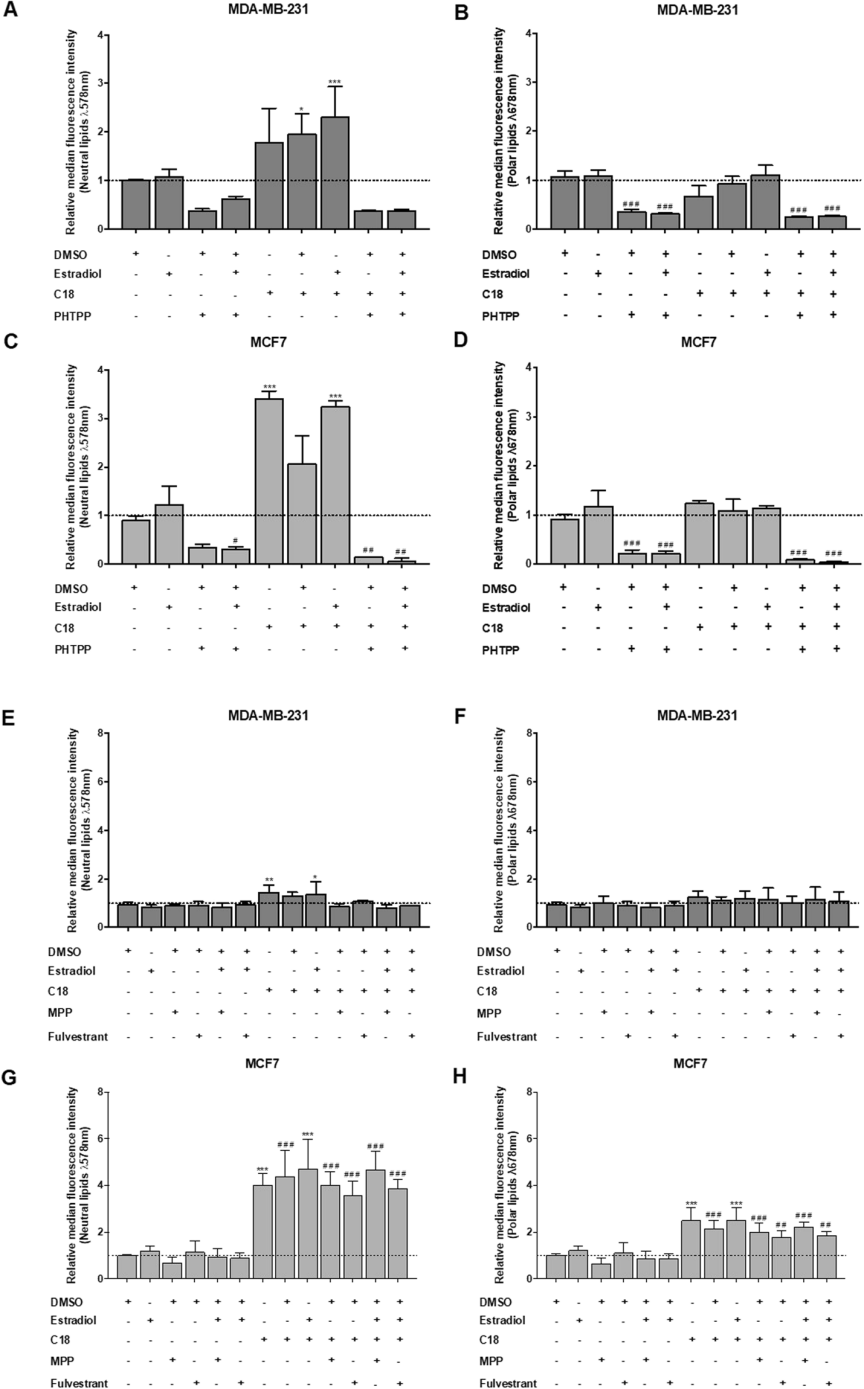
MDA-MB-231 and MCF7 cells exposed to PHTPP accumulate less FA. Cells were cultured in control (baseline culture medium represented by the dot line), control DMSO, estradiol and/or C18 conditions, in the presence or absence of PHTPP (**A–D**) or in the presence or absence of MPP and Fulvestrant (**E–H**). A flow cytometry analysis of Nile red staining was performed to evaluate the accumulation of neutral (λ 578 nm) and polar (λ 678 nm) lipids. Values are referenced to control/DMSO conditions within each cell line. Biological triplicates were tested, only adherent cells were analyzed and dead cells in culture media were discarded. Results are shown as mean ± SD. *p < 0.05, **p < 0.01, ***p < 0.001. (*) represents the statistical analysis in relation to control condition and (#) represents the statistical analysis in relation to control DMSO condition. One-way ANOVA with Dunnett’s multiple comparisons test.

To evaluate if the dynamics of FATP1 regulation, by estradiol, interfere with the FA uptake, a flow cytometry assay was performed using Nile Red labelling (Fig. 4). PHTPP, MPP and Fulvestrant were tested, with and without C18. MDA-MB-231 cells exposed to C18 plus estradiol showed a significant uptake of neutral lipids (Fig. 4A). When cells were exposed to PHTPP, the uptake of FA was decreased, being statistically significant in the polar lipids in both cell lines (Fig. 4A–D). C18 and estradiol in separate led to a higher uptake of neutral lipids in MCF7 cells. The effect of MPP and Fulvestrant in the FA uptake was also evaluated. These antagonists did not interfere with the FA uptake when comparing with the control conditions as shown in Fig. 4E–H. These findings indicate that ER-β is important in the uptake of FA, since its inhibition decreased the levels of FA. We can also observe that C18 in combination with estradiol is associated with an enhanced uptake of FA that might be transported by FATP1.

### Breast cancer cells (BCCs) viability is regulated by ER-β

**Figure 5:**
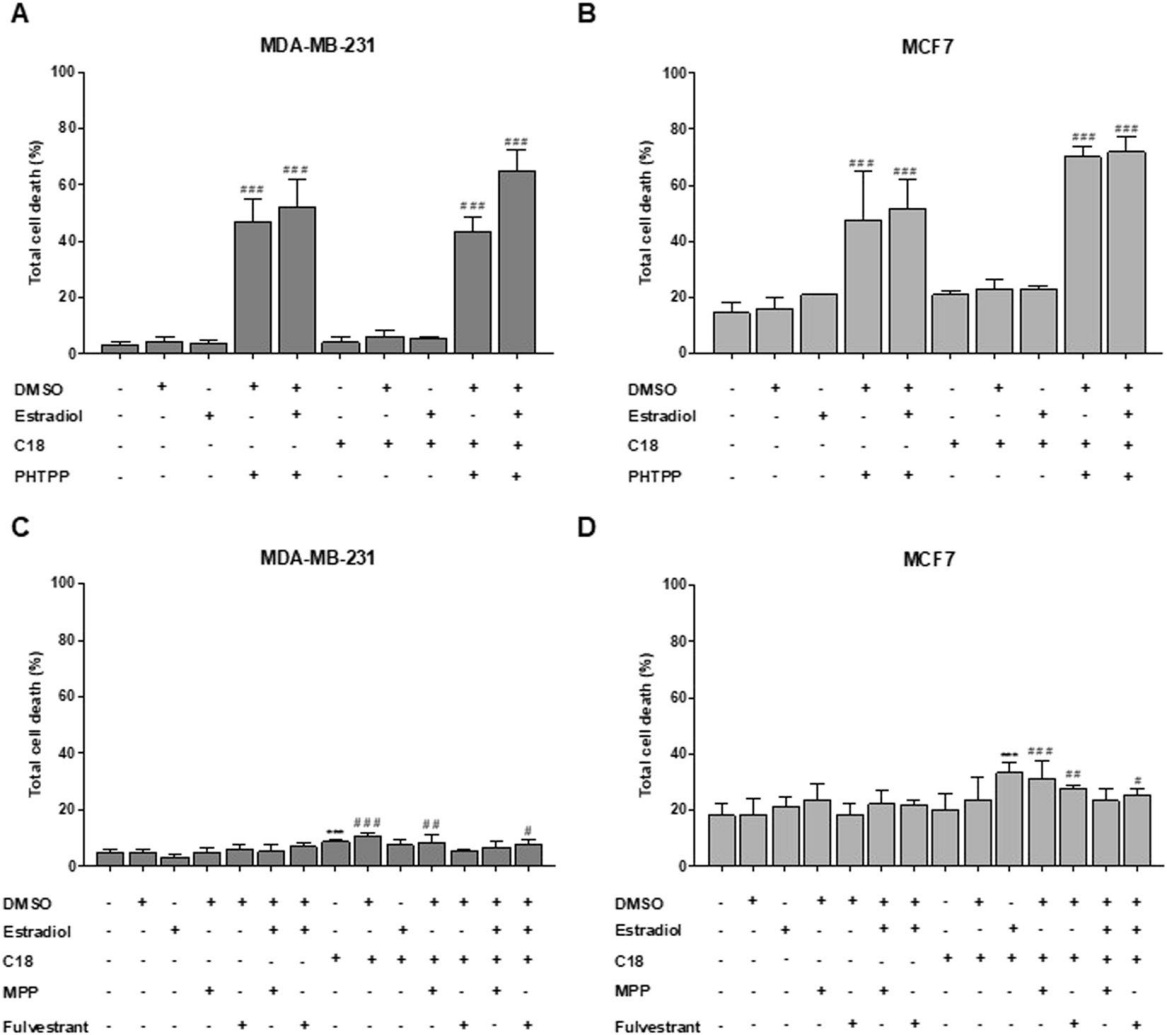
PHTPP affects cell viability of MDA-MB-231 and MCF7. Cells were cultured in control, control DMSO, estradiol and/or C18 conditions, in the presence or absence of PHTPP (**A**,**B**) or in the presence or absence of MPP and Fulvestrant (**C**,**D**) and were analyzed by flow cytometry. Results are shown as mean ± SD. *p < 0.05, **p < 0.01, ***p < 0.001. (#) represents the statistical analysis in relation to DMSO condition. Biological triplicates were tested, adherent cells were dead cells in culture media were analyzed. One-way ANOVA with Dunnett’s multiple comparisons test.

We performed a cell viability assay to evaluate the effect of PHTPP, an ER-β antagonist, in cells cultured in the presence or absence of estradiol with or without C18. Figures 5A,B show that PHTPP, with and without C18, caused a significant increase in cell death in both cell lines while the other conditions did not affect cell viability. Comparing with DMSO condition, MDA-MB-231 and MCF7 cells display similar levels of cell death upon exposure to PHTPP. These results indicate that ER-β plays a role in both cell lines survival.

Regarding MPP and Fulvestrant (ER-α antagonists), these compounds affected cell viability in a much lower extent than PHTPP (Fig. 5C,D). These results were expected for MDA-MB231 cell line because it is a TNBC cell line^22,23^. Regarding the assay testing the effect of MPP and Fulvestrant in MDA-MB-231, we observed that C18 and C18 plus estradiol induced slightly higher levels of cell death, though significant when compared with the control.

### Arylpiperazine 5k (DS22420314) impairs FA uptake and decreases cell viability

Since the results suggested that FA and estradiol, via ER-β, are relevant in the regulation of *FATP1/SLC27A1* and since this gene is upregulated in high grade BCs, mainly TNBC, we hypothesized that the pharmacological inhibition of FATP1 might be a strategy to fight cancer. To test this therapeutic approach, cells were exposed to arylpiperazine 5k (DS22420314)^24^, a compound that acts as a FATP1 inhibitor, interfering with the transport of FA across the cell membrane.

**Figure 6:**
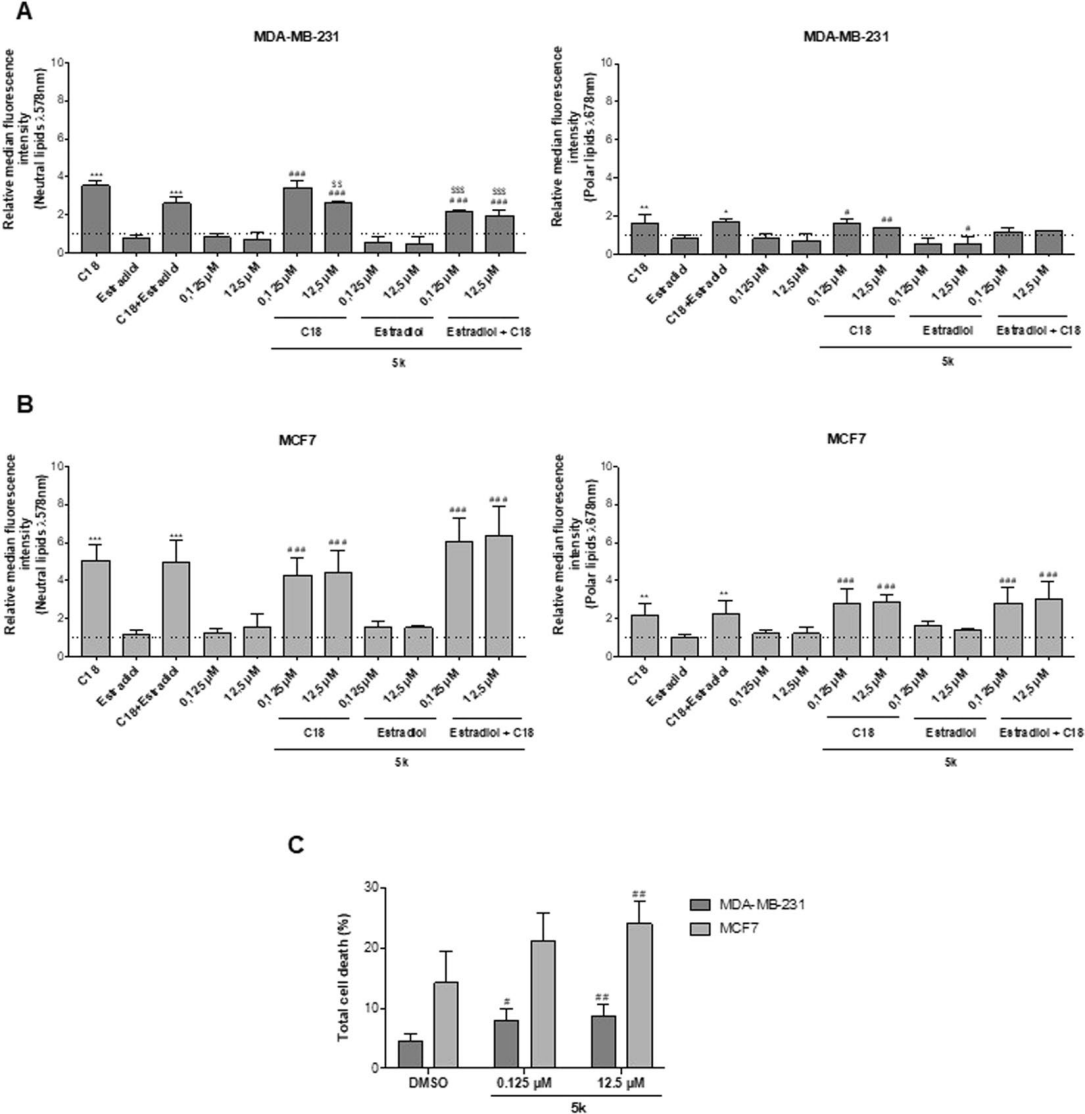
Arylpiperazine 5k (DS22420314) interferes with FA uptake and cell viability. Cells were cultured in control, control DMSO, estradiol with and without C18 and exposed to arylpiperazine 5k (DS22420314). Nile red labelling was analyzed by flow cytometry in MDA-MB-231 cells (**A**) and MCF7 cells (**B**). Cell death assay was performed in cells exposed to arylpiperazine 5k (DS22420314) and compared to control and control DMSO, using flow cytometry using Annexin V and PI (**C**). Values are referenced to control/DMSO conditions within each cell line. Data are means of biological triplicates. Results are shown as mean ± SD. *p < 0.05, **p < 0.01, ***p < 0.001. (*) represents the statistical analysis in relation to the control condition; (#) statistical analysis in relation to the DMSO condition; ($) statistical analysis in relation to the C18 condition. Biological triplicates were tested, adherent cells were dead cells in culture media were analyzed. The dot line defines the relative median fluorescence intensity for the control conditions. Multiple comparisons were performed using One-way ANOVA with Dunnett’s or Tukey’s test.

The uptake of FA in cells exposed to arylpiperazine 5k (DS22420314) (0.125 and 12.5 μM) was assessed by flow cytometry using Nile red, in MDA-MB-231 and MCF7 cells cultured in control, control DMSO and estradiol with and without C18. In Fig. 6, C18 exposure led to a significant increase in FA uptake in comparison with control cells (dot line), in both cell lines. MDA-MB-231 cells tended to accumulate less neutral and polar lipids when cultured both in the presence of estradiol and/or 5k (Fig. 6A). A significant decrease in FA uptake was observed in 5k exposed cells comparatively to cells that were only exposed to C18. It is of note that the higher concentration of 5k (12.5 μM) led to a lower uptake of neutral lipids, indicating a dose-dependent effect.

MDA-MB-231 exhibited a lower uptake of neutral lipids when cells were cultured in estradiol and 5k, in separate or in combination (Fig. 6A). In MCF7, when 5k was combined with C18 with and without estradiol an accumulation of neutral and polar lipids was observed (Fig. 6B). Thus, the treatment with arylpiperazine 5k (DS22420314) had an impact in lipid uptake in an environment rich in FA, mimicked by C18 supplementation, in MDA-MB-231 cells but not in MCF7 cells.

With the intent of analyzing the effect of arylpiperazine 5k (DS22420314) on cell viability, a cell death assay was carried out. In MDA-MB-231 cells, the total percentage of cell death was significantly higher in cells cultured in the two concentrations of 5k, comparing to control DMSO (Fig. 6C). In the MCF7 cell line, similar results were obtained but only the higher concentration of 5k showed significant higher cell death levels (Fig. 6C).

### Validation of results in additional breast cancer cell (BCC) lines

In order to confirm the reliability of results within BC context, we performed pivotal assays in two additional BCC lines (HCC1806, TNBC and BT-474, Luminal B).

**Figure 7:**
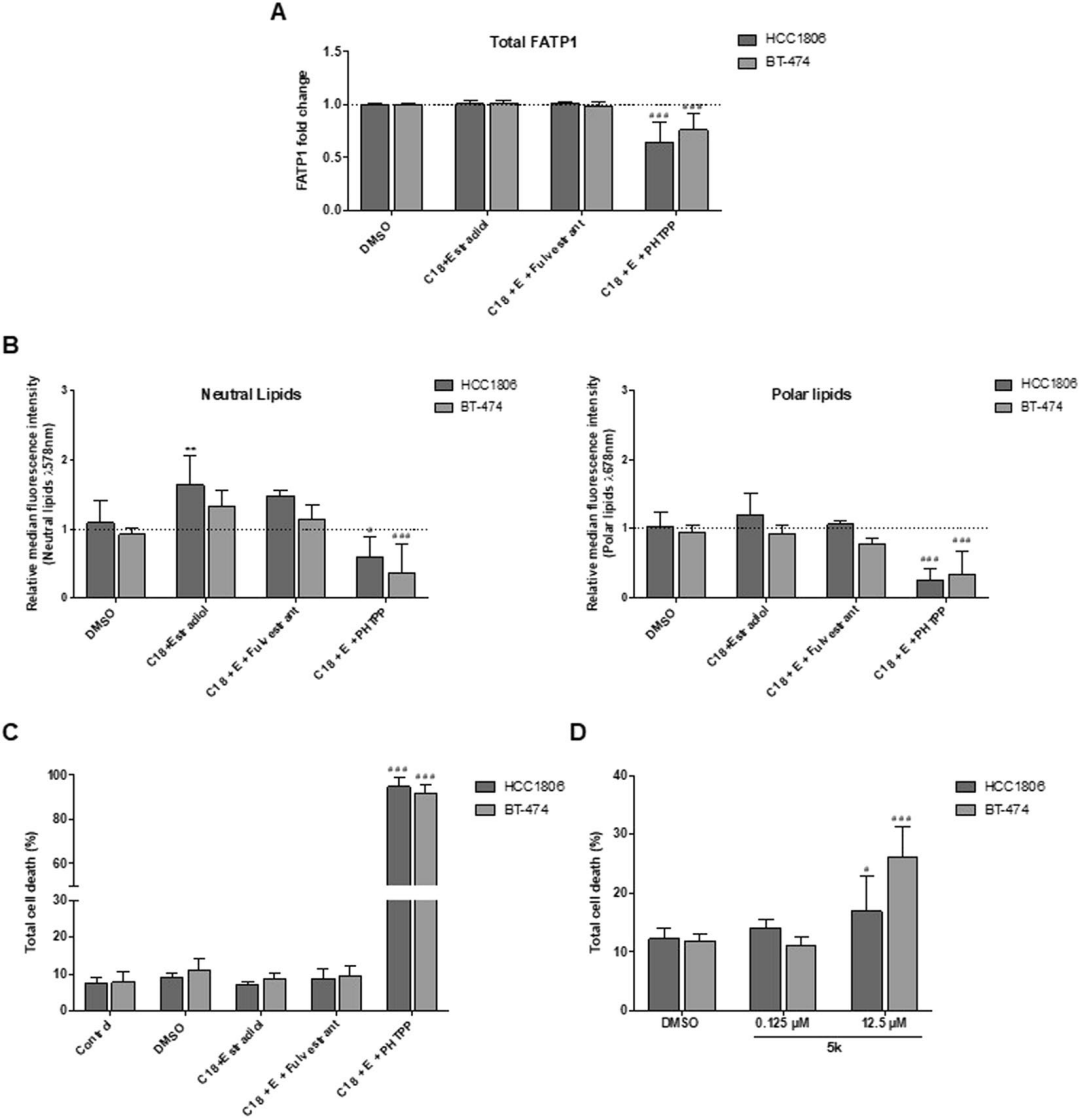
ER-β signaling regulates the expression of FATP1 and FA uptake in BT-474 and HCC1806 cells. BT-474 and HCC1806 cells were cultured in control, control DMSO, estradiol and C18 conditions, in the presence or absence of Fulvestrant and PHTPP (**A**–**C**). Cells were also exposed to arylpiperazine 5k and compared to control DMSO (**D**). Total *FATP1/SLC27A1* expression was analyzed by flow cytometry (a). Nile red labelling was analyzed to evaluate the accumulation of neutral (λ 578 nm) and polar (λ 678 nm) lipids by flow cytometry (**B**). Cell death assay was performed with flow cytometry using Annexin V and PI (**C**,**D**). Values are referenced to control/DMSO conditions within each cell line. Results are shown as mean ± SD. *p < 0.05, **p < 0.01, ***p < 0.001. (*) represents the statistical analysis in relation to control condition and (#) represents the statistical analysis in relation to DMSO condition. Biological triplicates were tested, only adherent cells were analyzed and dead cells in culture media were discarded; except in cell death analysis in which adherent and dead cells were analyzed together. Multiple comparisons were performed using One-way ANOVA with Dunnett’s or Tukey’s test.

The effect of the ER-β antagonist (PHTPP) and ER-α antagonist (Fulvestrant) was evaluated in these cells to validate the role of ER-β on *FATP1/SLC27 A1* expression. In both cell lines, the amount of total FATP1 positive cells was significantly reduced when ER-β was blocked with the addition of the antagonist PHTPP (Fig. 7A).

Regarding lipid uptake, a significant increase in neutral lipids was observed in HCC1806 cells when treated with C18 in combination with estradiol. No further differences were observed among conditions expect for the C18 plus estradiol and PHTPP condition showing a significant decrease in FA accumulation in both cell lines (Fig. 7B) corroborating our previous results.

Cell death analysis was also performed and once again the blockage of ER-β resulted in a significant increase in cell death while the other conditions did not affect cell viability suggesting that ER-β signaling supports BCCs survival (Fig. 7C). Fulvestrant did not affect neither FA accumulation nor cell viability, reinforcing the crucial role of ER-β in BCCs.

To explore the role of arylpiperazine 5k derivative (DS22420314) in cell viability, cells were exposed to two different concentrations of the drug (0.125 μM and 12.5 μM). Total cell death increased significantly in cells exposed to 12.5 μM of 5k (Fig. 7D). Arylpiperazines had a higher impact on BT-474 cells resulting in a greater cell death comparing to HCC1806.

This set of results is in agreement with our previous findings suggesting that ER-β plays an important role in FATP1 regulation and trafficking supporting cell survival.

## Discussion

Metabolic remodeling leads to changes in biosynthetic and bioenergetic pathways not only in neoplastic cells but also in non-cancerous cells in the same microenvironment^25^. This cellular and molecular network sustains the high demand for energy and biomass production which are essential for cancer initiation and progression^26^. In breast and ovarian cancer cells it was stated that tumor microenvironment promotes tumor growth and provides a rationale for the development of targeted therapies that block cancer metabolism fueled by the microenvironment^27,28^. Recently, our team^15^ has published that cancer associated fibroblasts (CAFs) cooperate with BCCs as FA suppliers, in *in vitro* and *in vivo* models, through the FA transporter FATP1. Aberrant lipid metabolism has long been recognized as a major metabolic event during cancer development^29^. However, most of the studies have been focused on *de novo* lipogenesis as the source for FA required for tumor cell growth, where the role of exogenous FA often disregarded^30–32^. The present work focuses on the uptake of exogenous FA by BCCs rather than on the *de novo* FA synthesis, considering the role of FATP1 as a mediator of FA transport. In addition, we also addressed ER-β as a regulator of *FATP1/SLC27A1* gene expression.

Most studies have been investigating FATP1 in the context of diet induced obesity, insulin resistance, hyperglycemia, hyperinsulinemia and hypertension^33–36^. To our knowledge, this is the first study that investigates the role of FATP1 in BC. Besides all the mechanistic experiments, our results also showed a statistically significant association between FATP1 expression and TNBC subtype, based on histochemistry analysis of breast tumors. Moreover, it was found that metabolic genes such as *SLC27* genes, including *FATP1/SLC27A1*, may be altered in metastatic cancer cells and are associated with poor prognosis^37^, as we observed in results from TCGA and GEO databases for BC. In other cancer contexts, an expression array analysis demonstrated that *FATP1/SLC27A1* expression is up-regulated *in vitro* and in human intrahepatic cholangiocarcinoma samples^38^. In addition, *FATP1/SLC27A1* mRNA was increased in rat hepatomas in comparison with normal liver tissue which correlated with FA uptake rates^39^. Accordingly, the FATP1 protein expression was evaluated in biopsies of patients with melanoma and it was found that 44% of samples overexpressed FATP1 specifically in the tumor cell compartment^40^.

Kaplan-Meier curves revealed that patients with upregulated expression of *FATP1/SLC27A1* showed lower overall survival (OS) and relapse free survival (RFS) in comparison with patients with low expression of *FATP1/SLC27A1* being even more apparent in more advanced stages of the disease (grade 3) (Fig. 1). Moreover, OS in grade 3 triple negative BC patients (TNBC) with high *FATP1/SLC27A1* expression was significantly lower than OS in grade 3, luminal A patients. These findings reveal that the *FATP1/SLC27A1* gene is undoubtedly relevant on the clinical outcome of BC as more aggressive and invasive carcinomas (TNBC) exhibit a significantly upregulated expression of *FATP1/SLC27A1*. These results were in agreement with the *in vitro* assays, since MDA-MB-231 cell line is from TNBC subtype. In that way, FATP1 can be considered as a marker for disease outcome. Interestingly and reinforcing the role of ER-β in the regulation of *FATP1/SLC27A1* expression, the TNBC group of tumors expressing high levels of *ESR2* showed low OS and the luminal A group showed high OS. Unfortunately, the software used did not allow the multivariate analysis in order to analyze the OS and the RFS in patients that co-express *ESR2* and *FATP1/SLC27A1*. The expression of ER-β and/or FATP1 is a promising way of identifying a subset of tumors in such a heterogeneous and indefinite subtype of BC, TNBC. A shortcoming in the analysis of BC databases is the fact that most of them are organized according to tumors staging and histological type and not the molecular subtype, which would be the element we intend to clarify in this paper issue. We believe the low number of cases classified according to the molecular subtype of BC is the main cause for the non-significant differences in RFS and OS between TNBC and luminal A cases. Nevertheless, the statistical significance in OS and RFS between TNBC grade 3 tumors expressing high levels *versus* low levels of *FATP1/SLC27A1*, is a strong indication that FATP1 plays a role in BC progression and outcome, suggesting that its usefulness as an outcome marker and therapeutic target deserves to be explored.

Our team have previously published that MDA-MB-231 exposed to CAFs exhibited a decreased FASN activity and an increased *FATP1/SLC27A1* transcriptional expression suggesting that BCCs change their metabolic profile from FA producers to FA gatherers^15^. Hence, our first experimental approach was to verify the expression of the *FATP1/SLC27A1* gene in MDA-MB-231 (TNBC), which is considered a highly aggressive and invasive cell line^22,23^. We also found that MDA-MB-231 cells exposed to C18 and estradiol showed higher *FATP1/SLC27A1* levels (Fig. 2A,B), suggesting that enhanced *FATP1/SLC27A1* expression is correlated with more aggressive phenotypes, being in agreement with the increased expression of *FATP1/SLC27A1* in TNBC subtype and the association with poor outcome of the disease (Fig. 1). Overall, the pulse chase assay showed that *FATP1/SLC27A1* mRNA levels are dynamic, with a significant increase after 30 min of C18 and C18 plus estradiol exposure, indicating that MDA-MB-231 cell line benefits from *FATP1/SLC27A1* expression in these conditions (Fig. 2A), to mediate the uptake of FA. Accordingly, cells exposed only to estradiol showed an accentuated and significant decrease of *FATP1/SLC27A1* mRNA with time (Fig. 2A). Because, without C18 boost, the stimulation of *FATP1/SLC27A1* expression by estradiol is not needed and negative feedback mechanisms may abrogate the expression of *FATP1/SLC27A1*. However, more detailed experiments on mRNA turnover mechanisms would be necessary. A very interesting observation was the levels of *ESR2* gene expression also in the pulse chase assay, showing that in MDA-MB-231 the highest levels of *ESR2* mRNA were achieved at 15 min after the stimulus with C18 plus estradiol (Fig. 2D), 15 min prior the significant increase of *FATP1/SLC27A1* mRNA levels (Fig. 2A). Also, in this cell line the expression of *ESR2* decreases over time in cells exposed to estradiol solely (Fig. 1C), showing synchrony between *ESR2* and *FATP1/SLC27A1* expression. The 15 min delay between the expression of *ESR2* and *FATP1/SLC27A1*, shows that *ESR2* expression is stimulated by estradiol in the presence of C18. *ESR2* behaves as an immediate early gene, whose expression is activated by a stimulus, and that afterwards will regulate the expression of *FATP1/SLC27A1*, a delayed early gene, that will contribute for the metabolic adaptive phenotype. This synergy is typical of the transmission of intracellular signal (from the cell membrane to the nucleus), requiring, as an answer, the expression of first line (immediate early) genes, usually transcription factors as ER-β, and second line (delayed early) genes, in order to fulfill a phenotype that will constitute the response to the initial stimulus^41^.

Our findings in four BC cell lines, suggest that ER-β is relevant in the modulation of *FATP1/SLC27A1* as well as a possible association with ER-β expression and mammary carcinogenesis. ER-α has been studied extensively in familial and sporadic BC but there is limited information on ER-β and its isoforms^9,42,43^. Studies have reported that ER-β2 expression was increased in invasive carcinomas in comparison with normal glands, in both ER-α−positive and ER-α-negative tumors^44^ and that cytoplasmic ER-β2 expression correlated with shorter overall survival at 15 years^42^. Moreover, an association of ER-β1 expression with increased cell proliferation in ER-α-negative BC was also described^45^. These evidences indicate that ER-β may have a role in the progression of BCs and could potentially serve as a prognostic marker. With the intent of investigating the role of ER-β in *FATP1/SLC27A1* regulation, cells were exposed to PHTPP, an ER-β antagonist^46^ and in order to exclude the interference of ER-α, cells were also tested with MPP^47^ and Fulvestrant^48^, ER-α antagonists. As expected, MDA-MB-231 was not affected by ER-α antagonists, since it is a TNBC cell line, and it was previously described that Fulvestrant does not affect this cell line viability^21^. The inhibition of ER-β strongly induced cell death while the inhibition of ER-α did not change cell death levels, suggesting the crucial role of ER-β in BCCs survival (Figs 5 and 7C).

Interestingly, in the assay testing the effect of MPP and Fulvestrant, we observed that C18 and C18 plus estradiol induced slightly higher levels of cell death in MDA-MB-231, though significant when compared with the control. This observation can be explained because One-way ANOVA analyzes the variance between different groups as a whole, and together Dunnett’s multiple comparisons test are used to compare each of a number of conditions/groups with a single control, considering the relative differences between all groups and finding the ones that are more different from the control^49,50^. Therefore, as the results for all the conditions in this assay are close to the control, slightly differences between control and C18 conditions are displayed as statistically significant. The same does not happen in the assay testing PHTPP, because there are conditions displaying much higher differences in cell death levels comparing to the control. However, a biological explanation can also be advanced, since we verified in other experiments that C18 and C18 plus estradiol induces an increased percentage of MDA-MB-231 cells in cell cycle S phase, meaning these conditions are stimulating cell proliferation (Supplementary Fig. 1). The increased proliferation rate is often accompanied by an increased cell death as mentioned by Gallaher *et al*.^51^.

In terms of lipid uptake, when cells were exposed to PHTPP, the uptake of FA was decreased in all cell lines (Figs 4A–D and 6B) whilst MPP and Fulvestrant did not interfere in the FA uptake (Fig. 4E–H). Hence, ER−β impairment targets the normal functioning of BCCs (Figs 3 and 6C) and leads to a decreased uptake of FA (Figs 4 and 6B).

Exploiting the features of cancer metabolism for cancer detection and treatment is a promising strategy in cancer therapeutics, diagnosis and prevention^52^. In many tumors like melanoma, breast and prostate cancer, lipids are provided via *de novo* lipogenesis, with an upregulation of FASN^53–55^. Inhibition of FASN by either small molecules (C75, orlistat) or small interfering RNA can efficiently suppress tumor cell growth *in vitro* and xenograft models^56–58^. Most FASN inhibitors show several adverse and side effects^59^, however our group has found that MDA-MB-231 exposed to CAFs exhibited a decreased FASN activity and an increased *FATP1/SLC27A1* expression^15^. Meaning that, depending on the microenvironment, increased FASN activity may not be a favorable adaptive mechanism if the cells are able to import FA. Accordingly, the pharmacological inhibition of FATP1 with arylpiperazine 5k (DS22420314)^24^ might be a strategy to fight cancer. We found that 5k significantly reduced the uptake of FA only in the TNBC cell line, MDA-MB-231 (Fig. 6A), indicating that the 5k compound might be a good inhibitor of FA uptake in cancer cells that express FATP1. The fact that normal cells retain the ability of producing FA^15,60^ will contribute for low adverse effects of this potential therapy. Regarding cell viability, the total percentage of cell death was significantly higher in cells cultured with 5k derivative, comparing to control DMSO (Figs 6C and 7D). Certainly, new experiments will be needed in order to validate and adjust therapeutic concentrations of arylpiperazine 5k (DS22420314) to treat cancer.

**Figure 8:**
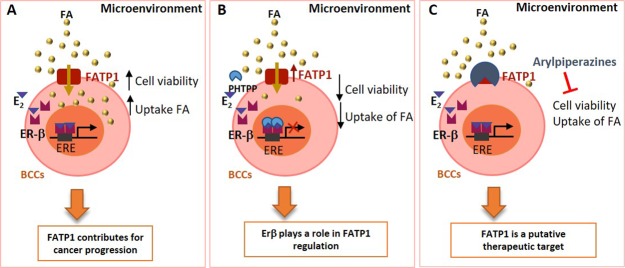
Stimulation of ER-β by estradiol contributes to *FATP1/SLC27A1* expression and cell survival of breast cancer cells (BCCs). FATP1, whose expression may be controlled by estradiol via ER-β that is a pro-survival element, mediating the transport of Fatty acids (FA), which are essential for BCCs (**A**). BCCs exposed to PHTPP (ER-β antagonist) show a decreased cell viability and lower uptake of FA suggesting that ER-β controls *FATP1/SLC27A1* expression (**B**). The inhibition of FATP1 with arylpiperazine 5k (DS22420314) interferes with the uptake of FA and cell viability indicating that FATP1 is a putative therapeutic target in BC (**C**).

The present study unraveled the crucial role of ER-β in *FATP1/SLC27A1* regulation and modulation in the uptake of FA (Fig. 8). Our *in vitro* findings are validated by patients’ data showing a higher expression of *FATP1/SLC27A1* in TNBC cases that conceptually more aggressive and invasive BCs. The inhibition of FATP1 with arylpiperazine 5k (DS22420314) interfered with the uptake of FA and cell viability, consistent with the importance of FATP1 as a putative therapeutic target in BC (Fig. 8). The meaning of ER-β positivity in BC is a matter that should be rescued for investigation.

## Materials and Methods

### Bioinformatics analysis

Data from Gene Expression Omnibus (GEO) and The Cancer Genome Atlas (TCGA) databases was used to analyze the gene expression of *FATP1/SLC27A1* in BC samples^18^. Kaplan-Meier curves of overall survival (OS) and recurrence-free survival (RFS) of BC patients were obtained from http://kmplot.com/analysis/.

### Morphology and immunohistochemistry analysis

Breast tumor samples were fixed in formaldehyde and embedded in paraffin. Sections (3 µm) were stained with hematoxylin and eosin staining (H&E) (Hematoxylin, Cat. Number CS700, Dako; and Eosin, Cat. Number CS701, Dako) and characterized by immunohistochemistry with anti–FATP1 antibody (Cat. Number MAB3304, R&D system, dilution 1:200 for 16 minutes; pretreatment CC1-24 min; Ventana Medical Systems, Tucson, Arizona, USA) on the BenchMark ULTRA IHC/ISH Automatic staining platform (Ventana Medical Systems) using OptiView DAB IHC Detection Kit (Ventana Medical Systems) with diaminobenzidine as the chromogen to detect antigen expression. Tissue sections were counterstained with Mayer’s hematoxylin before mounting. All antibody dilutions were made in Antibody Diluent Reagent Solution (Cat. Number 003218, Life Technologies). Image acquisition was performed in Digital Microimaging Device Leica DMD108 (version 1.15 Build 704, Leica Microsystems).

### Cell lines and culture conditions

Human cell lines from triple-negative BC (TNBC), MDA-MB-231 (HTB-26™, ATCC) and HCC1806 (CRL-2335™, ATCC); from luminal-A BC, MCF7 (HTB-22TM, ATCC), and from luminal B, BT-474 (HTB-20TM, ATCC), were obtained from American Type Culture Collection (ATCC, Manassas, VA, USA). Cells were maintained at 37 °C in a humidified environment of 5% CO2 in Dulbecco’s modified essential medium 1X (DMEM) (41965-039, Gibco, Life Technologies). Medium was supplemented with 10% fetal bovine serum (FBS; S 0615, Merck), 1% Antibiotic-Antimycotic (AA; P06-07300, PAN Biotech) and 50 µg/mL Gentamicin (15750-060, Gibco, Life Technologies). Cells were cultured until an optical confluence of 75–100% before they were detached with 0.05% trypsin-EDTA 1X (25300-054, Invitrogen). Before any *in vitro* experiment, cells were synchronized under starvation (FBS free culture medium), for 8 h. For experimental conditions cells were cultured in the presence and/or absence of FA (C18:2 - linoleic acid water-soluble, 96 µM; L5900, Sigma Aldrich; from now on called C18) and/or estradiol (1 nM; E4389, Sigma Aldrich) for 16 h. Cells were also exposed to estradiol (1 μM) and/or C18 (96 μM) and/or PHTTP (50 μM, ER-β antagonist; SML1355, Sigma Aldrich) and/or MPP (2 μM, ERα antagonist; M7068, Sigma Aldrich) and/or Fulvestrant (1 μM, ER-α antagonist; I4409, Sigma Aldrich) for 16 h. The concentrations of PHTTP, MPP and fulvestrant were selected according to the literature^19–21^. The inhibition of FATP1 was assessed by incubating cells with arylpiperazine 5k (DS22420314) (0.125 and 12.5 µM) for 16 h.

### Quantitative real-time PCR

RNA was extracted using RNeasy Mini Extraction kit (74,104, Qiagen, Hilden, Germany) and cDNA synthesized from 1 µg RNA and reversely-transcribed by SuperScript II Reverse Transcriptase (18080e44, Invitrogen), both according to the manufacturer’s protocol. Quantitative Real-Time PCR was performed using SYBR Green PCR Master Mix (04707516001, Roche), according to manufacturer’s protocol. Primers used were: *FATP1/SLC27A1* primer forward 5′- CAACATGGACGGCAAGGTC -3′ and primer reverse 5′- CAGCAGCTCCATTGTGTCCTC -3′; *ESR1* primer forward 5′- GCCAGGCTTTGTGGATTTGAC-3′ and primer reverse 5′- GGAGCAAACAGTAGCTTCCC-3′, and *ESR2* primer forward 5′- GATCTTGTTCTGGACAGGGATG-3′ and primer reverse 5′- GGAATTGAGCAGGATCATGGC -3′. Real-time PCR was carried out during 40 amplification cycles, according to manufacturer’s instructions, using a Lightcycler® 480 System instrument (05015243001, Roche). Hypoxanthine-guanine phosphoribosyltransferase (HPRT) was used as housekeeping gene (primer forward: 5′-TGACACTGGCAAAACAATGCA-3′; primer reverse: 5′-GGTCGTTTTTCACCAGCAAGCT-3′). Experiments were performed in biological triplicates and only the adherent cells were analyzed, cells in suspension were rejected.

### Western blotting

Cell pellets were lysed in Radio-Immunoprecipitation Assay (RIPA) buffer. Protein concentration was established by Bradford method, using Bio-Rad protein assay reagent (500–0006, Bio-Rad) through spectrophotometric quantification (595 nm). After protein quantification, loading buffer containing 10% SDS, 0.5% bromophenol blue in Tris-HCL (pH 6.8) and 10% β-mercaptoethanol (M3148, Sigma) was added to each cell lysate and boiled at 95–100 °C for 10 min. Total protein (50 µg) was loaded in 12% polyacrylamide gel (Tris-glycine SDS-Polyacrylamide gel) and electrophoresis was carried out in MINI-PROTEAN Tetra Electrophoresis System (Bio-Rad). After, proteins were transferred to an Immun-Blot® PVDF membrane with Trans-Blot® Turbo TM Blotting system. For protein detection, membranes were incubated with primary specific antibodies (mouse anti-human FATP1, 1:1000, MAB3304, R&D systems). Blots were further incubated with secondary antibody IgG-conjugated Horse-raddish peroxidase (HRP; anti-mouse, 1:5000, 31430, Thermo Scientific) and immunoreactive bands were detected by using ECL western blotting substrate (SuperSignal® West Pico Chemiluminescent Substrate (34080, Thermo Scientific) in a ChemiDoc XRS System (Bio-Rad) with Image Lab software. As endogenous control β-actin as used, membranes were re-incubated using mouse anti-human β-actin (1:5000, A5441, Sigma). Experiments were performed in biological triplicates and only the adherent cells were analyzed, cells in suspension were rejected.

### Chromatin immunoprecipitation (ChIP)

ChIP was employed to analyze putative interactions between ER-α and ER-β transcription factors and Estrogen Responsive Elements (ERE) sequences in *FATP1/SLC27A1* promoter. Those regions localize in 5′UTR (unstranslated reagion) of *FATP1/SLC27A1* gene between nucleotides −179 to −406. To crosslink proteins and DNA cells were treated with 37% formaldehyde at a final concentration of 1% (v/v), followed by an incubation with 125 mM glycine (pH 9). Subsequently, cells were scraped, centrifuged at 150 g for 2 min and ChIP lysis buffer (50 mM Tris-HCL (pH 8.0), 10 mM EDT, 1% SDS, 1 Complete, Mini, EDTA-free Protease Inhibitor Cocktail) was added. Cell lysates were sonicated and the size of the chromatin fragments (expected size between 1000 bp and 500 bp) was evaluated by electrophoresis, in a 1.2% (w/v) agarose gel. ChIP assay was performed using OneDay ChIP kit (kch-onedIP-060, Diagenode) according to the manufacturer’s protocol. The chromatin complexes were immunoprecipitated with 1 μl (~1 μg/mL) of specific antibodies: rabbit anti-human ER-α (ab75635; Abcam) and rabbit anti-human ER-β (ab196787; Abcam). Experiments were performed in biological triplicates and only the adherent cells were analyzed, cells in suspension were rejected. The relative occupancy of the immunoprecipitated factors at a specific promoter region was performed by RQ-PCR as described in section 4.2, using primers for *FATP1/SLC27A1* promoter (primer forward: 5′-GACTGTTGTAAGATTGGCAGGG-3′; primer reverse: 5′-CTGGGATTGGTCAACTCCTC-3′) and calculated using the following formula:

$${\rm{Relative}}\,{\rm{occupancy}}={2}^{({\rm{CtNegCtl}}-{\rm{CtTarget}})}$$

### FATP1 levels by flow cytometry

To evaluate the effects of estradiol, PHTPP, MPP and Fulvestrant, with and without FA in FATP1 expression FATP1 levels (total and membrane protein) were quantified using flow cytometry. Briefly, 1.5 × 10^5^ cells/mL were plated in 24-well plates and after starvation, cells were exposed to PHTPP (2-Phenyl-3-(4-hydroxyphenyl)-5,7-bis(trifluoromethyl)-pyrazolo[1,5-a] pyrimidine), MPP (1,3Bis(4-hydroxyphenyl)-4-methyl-5[4-(2-piperidinylethoxy)phenol]-1H-pyrazole-dihydrochloride) and Fulvestrant ((7α,17β)-7-[9-[(4,4,5,5,5-Pentafluoropentyl)sulfinyl] nonyl]estra-1,3,5(10)-triene -3,17-diol) with or without estradiol and FA, for 16 h. Cells were detached with PBS-EDTA, centrifuged at 230 g for 5 min, at 4 °C and washed with PBS-BSA 0.1% (v/w). Cells were incubated with 100 μL of FATP1 (1:500 in PBS-BSA 0.5%) with (total levels determination) and without (membrane levels determination) 0.1% saponin (v/w) (47036, Sigma Aldrich), for 1 h, at 4 °C. After, PBS-BSA 0.1% was added and cells were centrifuged at 230 g for 5 min, at 4 °C, followed by an incubation of 30 min with anti-mouse IgG- fluorescein (FITC) conjugated (1:1000 in in PBS-BSA 0.5%; A11059, Invitrogen), with and without 0.1% saponin. After, cells were washed, centrifuged and resuspended in 200 μL PBS-BSA 0.1%. Samples were analyzed in FACScalibur (Becton Dickinson) and data analyzed with FlowJo 8.7 software. Experiments were performed in biological triplicates and only the adherent cells were analyzed, cells in suspension were rejected.

### Cell death analysis by flow cytometry

After experimental conditions, supernatants were collected and cells were harvested with trypsin and centrifuged at 150 g for 2 min. The cells in suspension were joined to the cells in suspension (in the supernatants) in order to assess the real percentage of cell death. Cell pellets were stained with 0.5 μl Annexin V- fluorescein (FITC)- (640906, BioLegend) in annexin V binding buffer 1X and incubated at RT, in dark for 15 min. After incubation, samples were resuspended in 200 μl PBS 1 × 0.1% (v/w) BSA and centrifuged at 150 g for 2 min. Cells were resuspended in 100 μl of annexin V binding buffer 1 × 2.5 μl propidium iodide (PI, 50 μg/mL; P4170, Sigma-Aldrich) was added and samples were analyzed by flow cytometry (FACScalibur – Becton Dickinson). Sample data was analyzed using FlowJo 8.7 software (https://www.flowjo.com). Experiments were performed in biological triplicates.

### Nile red staining

Nile red staining was performed based on Greenspan *et al*. (1985) and Yao *et al*. (2011). For flow cytometry analysis, cells were detached with trypsin, washed PBS 1x and stained with Nile Red (1 µg/mL) for 15 min at room temperature. After incubation, cells were centrifuged, PBS 1X was added and samples were analyzed on FACSCalibur flow cytometer (Becton-Dickinson). Data was analyzed using FlowJo software (http://www.flowjo.com/). Experiments were performed in biological triplicates and only the adherent cells were analyzed, cells in suspension were rejected.

### Statistical analysis

Statistical analysis was performed using GraphPad Prism 6.0 software (www.graphpad.com). Sample data were presented as the mean (normal distribution) ± SD. Comparisons between data from each group were statistically analyzed by a two-tailed unpaired Student’s t-test and multiple comparisons were performed using One-way ANOVA with Dunnett’s or Tukey’s test. For pulse chase qPCR experiments of *FATP1/SLC27A1* mRNA quantification a Two-way ANOVA with Tukey’s test was used. Immunohistochemical data was analyzed using univariate analysis (two-tailed t-test) on SPSS software. Differences between experimental conditions were considered statistically significant at *p* < 0.05. Experiments were performed in biological triplicates.

## Supplementary information

supplemmentary data
