# Supplementary material for: Unraveling FATP1, regulated by ER-β, as a targeted breast cancer innovative therapy
Source: Sci Rep. 2019 Oct 1;9:14107. doi: 10.1038/s41598-019-50531-3 (PMC6773857; doi:10.1038/s41598-019-50531-3)
Supplement: Supplementary file 1 — supplemmentary data [file 41598_2019_50531_MOESM1_ESM.pdf]

Unraveling FATP1, regulated by ER-β, as a targeted breast cancer innovative therapy

Cindy Mendes,[1,2] Filipa Lopes-Coelho,[1,2] Cristiano Ramos,[1,2] Filipa Martins,[1,2] Inês Santos,[1,2] Armanda Rodrigues,[1,2] Fernanda Silva,[1,2] Saudade André,[1,2] Jacinta Serpa[1,2*]

[1]CEDOC, Chronic Diseases Research Centre, NOVA Medical School|Faculdade de Ciências Médicas, Universidade NOVA de Lisboa, Campo dos Mártires da Pátria, 130, 1169-056 Lisboa, Portugal

[2]Instituto Português de Oncologia de Lisboa Francisco Gentil (IPOLFG), Rua Prof Lima Basto 1099-023, Lisboa, Portugal

*Correspondence: jacinta.serpa@nms.unl.pt; Tel.: +351217229818

Cindy Mendes; E-mail: cindymendes8@gmail.com

Filipa Lopes-Coelho; E-mail: filipa.coelho@nms.unl.pt

Cristiano Ramos; E-mail: ramos.cristiano.93@gmail.com

Filipa Martins; E-mail: filipamardias@msn.com

Inês Santos; E-mail: ips.ines95@gmail.com

Armanda Rodrigues; E-mail: maria.rodrigues@nms.unl.pt

Fernanda Silva; E-mail: fernanda.silva@nms.unl.pt

Saudade André; E-mail: sandre@ipolisboa.min-saude.pt

*Jacinta Serpa; E-mail: jacinta.serpa@nms.unl.pt

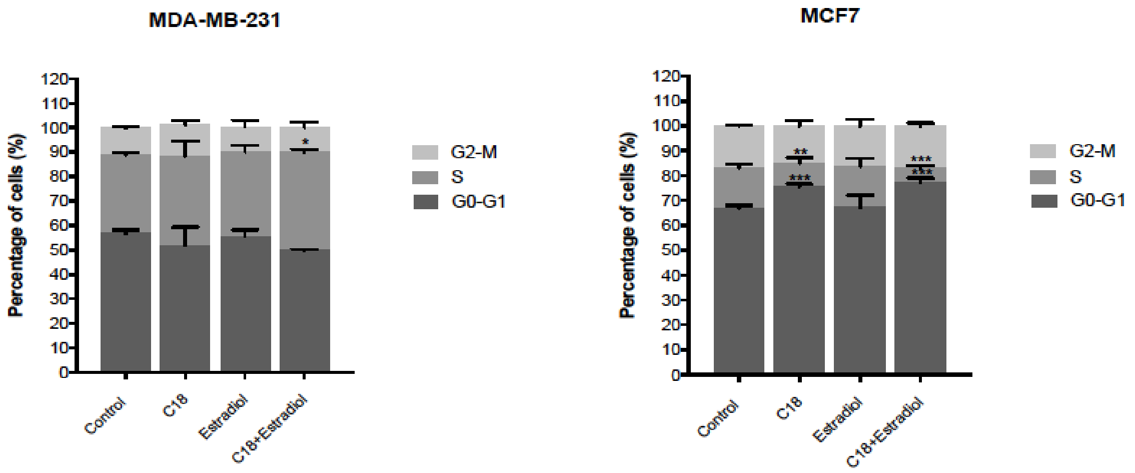

Supplementary Figure 1. Fatty acids (C18) stimulate proliferation in MDA-MB-231 but not in MCF7 cell line. Cells were cultured in the presence and/or absence of FA (C18:2 - linoleic acid water-soluble, 96 µM; L5900, Sigma Aldrich; from now on called C18) and/or estradiol (1 nM; E4389, Sigma Aldrich) for 16h. Cell cycle was analyzed by flow cytometry, using 70% ethanol fixed cells and propidium iodide (PI) labelling. Differences between experimental conditions were considered statistically significant at $p<0.05$. (*) represents the statistical analysis in relation to control condition.
